# Supplementary figures and images for: Using molecular characteristics to distinguish multiple primary lung cancers and intrapulmonary metastases
Source: PeerJ. 2024 Jan 31;12:e16808. doi: 10.7717/peerj.16808 (PMC10838092; doi:10.7717/peerj.16808)

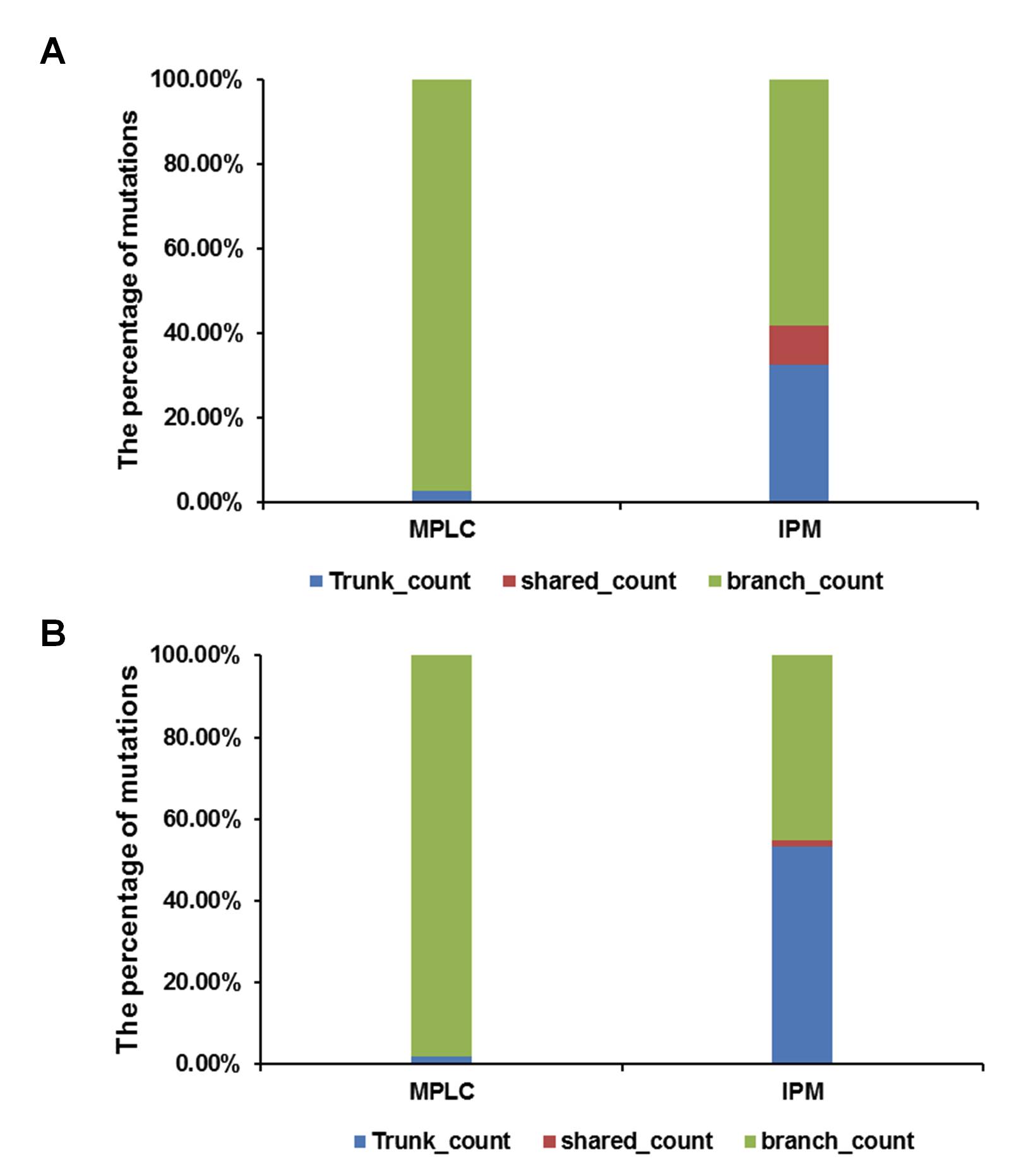

Supplement: Supplemental Information 1 — (A) The ratio of trunk, shared, and branch mutations are shown in MPLC and IPM of training cohort. (B) The ratio of trunk, shared, and branch mutations are shown in MPLC and IPM of validating cohort. [file peerj-12-16808-s001.jpg]
