# Supplementary material for: Using molecular characteristics to distinguish multiple primary lung cancers and intrapulmonary metastases
Source: PeerJ. 2024 Jan 31;12:e16808. doi: 10.7717/peerj.16808 (PMC10838092; doi:10.7717/peerj.16808)
Supplement: Supplemental Information 2 [file peerj-12-16808-s002.docx]

Supplementary Table 1. Genetic alterations and histological types of each tumor obtained from multiple lung cancer patients of training cohort.

| Patient | Histological  type | Number of matching  mutations | Matching genes | |  |
| --- | --- | --- | --- | --- | --- |
| TP1 |  |  |  | |  |
| T1 | ADC | None | ___ |  |  |
| T2 | ADC |  |  |  |  |
| TP2 |  |  |  | |  |
| T1  T2  T3 | SCC  SCC  SCC | T1 and T2=1,  T1 and T3=3  T2 and T3=1 | NTRK1 p.G137V，NTRK1 c.288-3C>A，PIK3CA p.K111N;  NTRK1 c.288-3C>A;  NTRK1 p.G137V，NTRK1 c.288-3C>A，PIK3CA p.K111N | |  |
| TP3 |  |  |  | |  |
| T1 | ADC | 3 | EGFR p. 746_T751del, GFR p. 790M, P53 p. 273L | |  |
| T2 | ADC |  |  |  |  |
| TP4 |  |  |  | |  |
| T1  T2  T3  T4 | C-SCLC  C-SCLC  C-SCLC  C-SCLC |  | CCNE1 cn_amp, CDH1 p. N56S, EGFR cn_amp, EGFR P. E746_A750del, KMT2D p. A2094I, NFE2L2 p. T260Pfs*8, RB1 p. F755V, SPTA1 p. R1811*, STK24 cn_amp, TOP1 p. I377V, TP53 p. Y220H, CDH1 p. N56S; | |  |
|  |  |  |  | |  |
|  |  | T1 and T2=9  T1 and T3=11  T1 and T4=2  T2 and T3=9  T2 and T4=2  T3 and T4=3 | EGFR cn_amp, EGFR p. E746_A750del, KMT2D p. A2094I, NFE2L2 p. T260Pfs*8, RB1 p. F755V, SPTA1 p. R1811*, STK24  cn_amp, TOP1 p. I377V, TP53 p. Y220H; | |  |
|  |  |  | CCNE1 cn_amp, CDH1 p. N56S, EGFR cn_amp, EGFR p. E746_A750del, KMT2D p. A2094I, NFE2L2 P. T260Pfs*8, PDGFRA p. D1075N, RB1 p. F755V, SPTA1 p. R1811*, STK24 cn_amp, TOP1 p. I377V, TP53 p. Y220H;  EGFR p. E746_A750del, PDGFRA p. D1075N, TP53 p. Y220H | |  |
| TP5 |  |  |  | |  |
| T1 | ADC | 2 | ERBB2 c.1022-6C>T, ROS1 fusion | |  |
| T2 | ADC |  |  |  |  |
| TP6 |  |  |  | |  |
| T1 | ADC | 1 | KRAS p. G13D | |  |
| T2 | ADC |  |  |  |  |
| TP7 |  |  |  | |  |
| T1 | ADC | 3 | EGFR cn_amp, EGFR p. L747_T751del, TP53 p. G244Afs*3 | |  |
| T2 | NSCLC |  |  |  |  |
| TP8 |  |  |  | |  |
| T1 | ADC | 2 | EGFR p. L858R, TP53 p. W146* | |  |
| T2 | ADC |  |  |  |  |
| TP9 |  |  |  | |  |
| T1 | ADC | 1 | EGFR p. L858R | |  |
| T2 | ADC |  |  |  |  |
| TP10 |  |  |  | |  |
| T1 | SCC | None | __ | |  |
| T2 | ADC |  |  |  |  |
| TP11 |  |  |  | |  |
| T1 | AIS | None | KRAS p. G12D; | |  |
| T2 | AIS |  | WT; | |  |
| TP12 |  |  |  | |  |
| T1 | AIS | None | __ | |  |
| T2 | MIA |  |  |  |  |
| TP13 |  |  |  | |  |
| T1 | ADC | None | __ | |  |
| T2 | ADC |  |  |  |  |
| TP14 |  |  |  | |  |
| T1 | ADC | 3 | EGFR p. L858R, PIK3CA p. N107I, TP53 c.920-1G>C | |  |
| T2 | ADC |  |  |  |  |
| TP15 |  |  |  | |  |
| T1 | ASC | All | EGFR p. T790M, EGFR p. L858R | |  |
| T2 | ASC |  |  |  |  |
| TP16 |  |  |  | |  |
| T1 | ADC | None | __ | |  |
| T2 | ADC |  |  |  |  |
| TP17 |  |  |  | |  |
| T1  T2 | ADC  ADC | 7 | FH p. A70P, GLI3 p. G457*, HDAC9 c.265-4C>A, LRP1 p. N2221S, MUC16 p. T10606K, SF3B1 c.28+7G>C, TP53 c.993+1G>T | |  |
|  |  |  |  |  |  |
| TP18 |  |  |  | |  |
| T1 | ADC | None | __ | |  |
| T2 | ADC |  |  |  |  |
| TP19 |  |  |  | |  |
| T1  T2 | ADC  LC | 21 | ALK p.T1087I, ARHGEF10 p.G118C, ARID2 p.M456I, BCORL1 p.G1357A, CD1D p.A11E, CREB3L2 p.S443N, CREBBP c.4728+8C>T, DIS3 c.1503+9A>G, DIS3L2 p.V113L, EPHA5 p.G18S, GALNT12 p.P240L, LRP1B c.344-9dup, NTRK2 c.213-7del, OBSCN p.T1956S, PARP1 p.A220V, PIK3C2B p.C691W, PKN1 c.1A>C, RELA c.1033+6del, SHQ1 p.I377M, TNFAIP3 p.P714S, XRCC3 p.D186N | |  |
|  |  |  |  |  |  |
| TP20 |  |  |  | |  |
| T1 | ADC | None | __ | |  |
| T2 | ADC |  |  |  |  |
| TP21 |  |  |  | |  |
| T1 | SCC | None | __ | |  |
| T2 | ADC |  |  |  |  |
| TP22 |  |  |  | |  |
| T1 | ADC | None | __ | |  |
| T2 | ADC |  |  |  |  |
| T3 | ADC |  |  |  |  |

TP1-22: Training patient 1-22; AIS: adenocarcinoma in situ; MIA: minimally invasive adenocarcinoma; IAC: invasive adenocarcinoma; SCC: squamous-cell carcinoma; ADC: adenocarcinoma; LC: lung carcinoid; C-SCLC: Compound small cell lung cancer; NSCLC: Non-small cell lung cancer; ASC: adenosquamous carcinoma of the lung.

Supplementary Table 2. Genetic alterations and histological types of each tumor obtained from multiple lung cancer patients of validating cohort.

| Patient No. | Histological  type | Number of matching  mutations | Matching genes |  |
| --- | --- | --- | --- | --- |
| VP1 |  |  |  |  |
| T1 | ADC | 2 | CDK4 cn_amp, EGFR p. L858R |  |
| T2 | ADC |  |  |  |
| VP2 |  |  |  |  |
| T1  T2 | ADC  ADC | 10 | EGFR cn_amp, EGFR p. E746_A750del, GNAS cn_amp, NPM1 cn_amp, RB1 rearrangement, SDHA cn_amp, TP53 p. G266E, TP53 p.C229*, TRIO cn_amp, ZNF217 cn_amp |  |
|  |  |  |  |  |
| VP3 |  |  |  |  |
| T1 | ADC | T1 and T2=4  T1 and T3=4  T2 and T3=6 | ABL2 p. P819A, CFTR p. I1000*, EGFR p. L858R, TP53 p. H179R; |  |
| T2 | ADC |  | ABL2 p. P819A, CFTR p. I1000*, EGFR p. L858R, TP53 p. H179R, FANCM p. I633M, NFKBIA cn_amp; |  |
| T3 | ADC |  | ABL2 p. P819A, CFTR p. I1000*, EGFR p. L858R, TP53 p. H179R, FANCM p. I633M, NFKBIA cn_amp |  |
| VP4 |  |  |  |  |
| T1 | IAC | None | __ |  |
| T2 | MIA |  |  |  |
| VP5 |  |  |  |  |
| T1 | AIS | None | __ |  |
| T2 | IAC |  |  |  |
| VP6 |  |  |  |  |
| T1  T2 | ADC  ADC | 39 | AFF3 p. N905S, AKT2 cn_amp, ARHGEF17 c.3891+3A>G, BRCA2 p. A1193T, CALR p. L367Tfs*46, CFTR p. E379Q, CYP17A1 p. F53L, CYP2D6 p. [F120I;A122S], DICER1 p. P623A, DLC1 p. R338H, EPHA7 p. E636K, EPHA7 c.1924+12G>T, EPHB1 p. K194Q, ERCC4 c.973+11A>T, FAM135B p. D415Y, FANCD2 p. D844H,FAT1 p.S3198F, GNA13 p.R140K, GTF2I c.1554-11T>C, KDM5A p.S1277C, KMT2C p.M2304I, LRP1B c.463+1G>T, LRP1B p.I2400T, OBSCN c.6487+4C>T, OBSCN p.E3680Q, PDGFRA c.49+10C>A, PREX2 c.3505-11A>G, PRKDC p.G5A, ROS1 p.D2213E, SF3B1 c.1720-11_1720-8del, SF3B1 p.R1262T, SMAD4 c.1140-9C>G, TP53 p.A70Vfs*53, TP63 p.Q380*, TYK2 p.P440S, WNK1 p.S2100F, ZFHX3 p.Q3204dup, ZFHX3 p.G131W, ZNF217 p.D566G |  |
|  |  |  |  |  |
| VP7 |  |  |  |  |
| T1 | IAC | None | __ |  |
| T2 | MIA |  |  |  |
| T3 | ADC |  |  |  |
| VP8 |  |  |  |  |
| T1 | AIS | 1 | EGFR p. L858R |  |
| T2 | ADC |  |  |  |
| T3 | ADC |  |  |  |
| VP9 |  |  |  |  |
| T1 | AIS | 1 | MAP2K1 p. E102_I103del |  |
| T2 | AIS |  |  |  |
| VP10 |  |  |  |  |
| T1 | AIS | None | __ |  |
| T2 | MIA |  |  |  |
| VP11 |  |  |  |  |
| T1 | AIS | None | __ |  |
| T2 | MIA |  |  |  |
| VP12 |  |  |  |  |
| T1 | AIS | None | __ |  |
| T2 | MIA |  |  |  |
| VP13 |  |  |  |  |
| T1  T2 | ADC  ADC | 12 | EMSY p. E1142*, FANCL c.96+11A>T, GLI3 p. E1076_M1080del, GNAS p. K214N, KRAS p. G12C, LRP1B p. G4593V, RBM10 p. W658C, ROCK1, p.H936N, SPEN p. P3080L, STK11 p. L117*, STK11 p. D194Y, TET1 p. S1924F |  |
|  |  |  |  |  |

AIS: adenocarcinoma in situ; MIA: minimally invasive adenocarcinoma; IAC: invasive adenocarcinoma; SCC: squamous-cell carcinoma; ADC: adenocarcinoma;

Supplementary Table 3. Molecular characteristics of patients with multiple lung cancer according to the final classification.

| Variables | MPLC (n = 21) | IPM (n = 14) | P value |
| --- | --- | --- | --- |
| EGFR status |  |  |  |
| WT | 10 (47.62%) | 4 (28.57%) | 0.31 |
| Mut | 11 (52.38%) | 10 (71.43%) |  |
| KRAS status |  |  |  |
| WT | 12 (57.14%) | 13 (92.86%) | 0.03 |
| Mut | 9 (42.86%) | 1 (7.14%) |  |
| TP53 status |  |  |  |
| WT | 12 (57.14%) | 6 (42.86%) | 0.50 |
| Mut | 9 (42.86%) | 8 (57.14%) |  |
| BRAF status |  |  |  |
| WT | 18 (85.71%) | 14 (100%) | 0.26 |
| Mut | 3 (14.28%) | 0 (0.00%) |  |
| ERBB2 status |  |  |  |
| WT | 16 (76.19%) | 12 (85.71%) | 0.68 |
| Mut | 5 (23.81%) | 2 (14.29%) |  |
| MET status |  |  |  |
| WT | 20 (95.24%) | 14 (100%) | 1 |
| Mut | 1 (4.76%) | 0 (0.00%) |  |
| NRAS status |  |  |  |
| WT | 20 (95.24%) | 13 (92.86%) | 1 |
| Mut | 1 (4.76%) | 1 (7.14%) |  |
| ALK status |  |  |  |
| WT | 19 (90.48%) | 13 (92.86%) | 1 |
| Mut | 2 (9.52%) | 1 (7.14%) |  |
| RET status |  |  |  |
| WT | 17 (80.95%) | 14 (100%) | 0.13 |
| Mut | 4 (19.05%) | 0 (0.00%) |  |
| ROS1 status |  |  |  |
| WT | 19 (90.48%) | 13 (92.86%) | 1 |
| Mut | 2 (9.52%) | 1 (7.14%) |  |
